# A Qualitative Study of How Adolescents’ Use of Coping Strategies and Support Varies in Line With Their Experiences of Adversity

**DOI:** 10.1007/s10566-022-09682-0

**Published:** 2022-03-01

**Authors:** Emily Stapley, Sarah Stock, Jessica Deighton, Ola Demkowicz

**Affiliations:** 1 Evidence Based Practice Unit (EBPU), Anna Freud National Centre for Children and Families and University College London (UCL), 4-8 Rodney Street, N1 9JH London, England; 2 Manchester Institute of Education, The University of Manchester, Manchester, England

**Keywords:** Adolescence, Coping, Support, Qualitative, Prevention, School

## Abstract

**Background:**

Adolescence is associated with a rise in the incidence of mental health issues. Thus, the factors, processes, and contexts that protect and promote positive mental health in adolescence are of key interest to policymakers.

**Objective:**

Our aim was twofold: First, to explore the coping strategies and sources of support that adolescents identify as protective (or not) in the face of difficulty over a three-year period; second, to examine how and why this may vary in line with the levels of adversity that they report experiencing in life.

**Methods:**

Participants were attending schools in England implementing a mental health prevention programme called HeadStart. 93 semi-structured interviews were conducted with 31 adolescents (age 11–12 at the outset of the study; 58% female) once per year over three years. The interviews were analysed using thematic analysis.

**Results:**

Six coping strategy themes (e.g., ‘Disengaging from problems’) and five support themes (e.g., ‘Parents as a source of comfort and advice’) were derived from the interviews. The types, quality, and consistency of reported coping strategies and support varied in line with whether adolescents were experiencing higher or lower levels of adversity in life over time, and according to the resources that they had available within their physical and social contexts.

**Conclusions:**

Our findings underscore the importance for mental health prevention programmes of bolstering both individual-level coping strategies and the resources available within adolescents’ environments to help them to manage adversity.

Adolescence is a period of major life change, characterised by physical transformations, psychological and cognitive development, and changes to peer and family relationships (Blakemore, 2012). Adolescence is also associated with a rise in the incidence of mental health issues, with the latest statistics in the UK indicating that among 11- to 16-year-olds, 17.6% had a diagnosable mental disorder in 2020, as compared to 14.4% of 5- to 10-year-olds (Vizard et al., 2020). It has been calculated that the cost of ‘late intervention’ to combat the problems that young people experience, such as mental disorders, is nearly £17 billion (Chowdry & Fitzsimons, 2016). Therefore, developing effective early intervention programmes, and ascertaining the factors, processes, and contexts that protect and promote adolescent wellbeing and positive mental health, is of key interest for policymakers seeking to prevent the onset of mental health issues in adolescence.

Researchers have distinguished between protective factors, which are associated with positive outcomes in the face of risk and adversity, and promotive factors, which are associated with positive outcomes generally (Masten & Barnes, 2018). The study of such factors features prominently in research seeking to explain why some individuals show resilience in the face of trauma, adversity, and risk, whereas others show poorer outcomes (e.g., Luthar, 2015; Masten & Barnes, 2018). Resilience can be broadly defined as the complex and dynamic process of adaptation to adversity (Luthar, Cicchetti, & Becker, 2000; Masten, 2014; Ungar, 2012), though we note that there are various subtleties and variations in how resilience can be understood (for an overview, see Southwick, Bonanno, Masten, Panter-Brick, & Yehuda, 2014).

Recent theory and research in this area has increasingly focused upon the embedded nature of resilience, whereby adaptation is facilitated through interactions between the individual and aspects of their ecological environment. For example, Ungar (2008) has defined resilience as a process whereby individuals navigate towards the resources to sustain their wellbeing that are available to them within their physical and social contexts. Thus, rather than putting the onus solely on the individual’s ability to cope, this definition underscores the role of both the individual and their environment in promoting wellbeing (Ungar, Brown, Liebenberg, Cheung, & Levine, 2008). Similarly, Masten (2021), advocating for a systemic perspective on resilience, has argued that the degree to which young people are able to respond adaptively in the face of disaster depends on the resilience of the interconnected systems around them, including family, school, community, and policy. Such definitions are underpinned by Bronfenbrenner’s (1979) ecological systems theory, which emphasises the role in child development of the child’s interaction with the interrelated, nested systems around them (Ungar, Ghazinour, & Richter, 2013).

Following early pioneers in the study of resilience (e.g., Garmezy, 1974, 1985), researchers have tended to distinguish between three broad categories of protective factors: individual factors, such as effective coping skills or high self-esteem; family factors, such as a positive caregiver-child relationship or family climate; and environmental or community factors, such as prosocial peers or a positive school environment (e.g., Eriksson, Cater, Andershed, & Andershed, 2010; Fritz, de Graaff, Caisley, van Harmelen, & Wilkinson, 2018; Olsson, Bond, Burns, Vella-Brodrick, & Sawyer, 2003). Thus, the concepts of coping and social support have prominence within the study of protective factors. Coping can be defined as the *“constantly changing cognitive and behavioral efforts to manage specific external and/or internal demands that are appraised as taxing or exceeding the resources of the person”* (Lazarus & Folkman, 1984, p.141), and social support as the resources that the individual’s social network provides to help them to handle difficulties (Cohen, 2004).

Research investigating protective factors has often been quantitative in design. For instance, numerous studies have examined what factors protect young people in the face of adversity (e.g., Askeland et al., 2020; Eriksson et al., 2010), which factors reduce the likelihood of young people developing mental health issues (e.g., Fritz et al., 2018; Fritz, Stochl, Goodyer, van Harmelen, & Wilkinson, 2020), which factors predict resilience following trauma (e.g., Lai, Lewis, Livings, La Greca, & Esnard, 2017; Masten, 2021), and in what ways the impact of protective factors varies by the level of adversity experienced (e.g., Bowen, Lee, & Weller, 2007; Kassis, Artz, Scambor, Scambor, & Moldenhauer, 2013). However, quantitative research in this area has been criticised for its lack of attention to how, why, and when particular factors, or combinations of factors, may be more or less protective for young people from their own perspectives and in their own words (Eriksson et al., 2010; Ungar, 2003). Qualitative research designs are well suited for answering such questions, including offering greater nuance in understanding the complex protective processes that are ecologically embedded within each individual’s world.

Previous qualitative studies have explored young people’s identification of the protective factors and processes that contribute to resilience in the context of academic attainment (e.g., Chee, 2019; Morales, 2008), economic disadvantage (Smokowski & Reynolds, 1999), and specific mental health difficulties (e.g., Everall, Altrows, & Paulson, 2006; Las Hayas et al., 2016), as well as young people’s ways of coping with adversity or stress in daily life (e.g., Stapley, Demkowicz, Eisenstadt, Wolpert, & Deighton, 2020a; Ungar et al., 2008). For example, through interviews with 13 young adults in Canada who overcame suicidality in adolescence, Everall et al. (2006) identified four domains of resilience: (a) social processes - having consistent, supportive relationships with others (such as family members, peers, teachers, and professionals); (b) emotional processes - being aware of and able to express feelings; (c) cognitive processes - gaining new perspectives and having a sense of control; and (d) taking action with purpose and specific goals in mind. In another Canadian study, Ungar et al. (2008) identified seven experiences that 19 adolescents described as enhancing their mental health, which they each had varying access to within their environments: material resources; supportive relationships; a desirable sense of self; a sense of power and control; cultural traditions; a meaningful role within the community; and feeling part of something bigger.

By illuminating protective factors and processes, and exploring how and why they may vary by context, resources, or the level of adversity experienced, qualitative research findings can inform the development of interventions seeking to bolster young people’s resilience and prevent the onset of mental health issues (Eriksson et al., 2010; Luthar, 2015). For instance, Ungar, Hadfield, and Ikeda (2018) interviewed 85 adolescents in Canada, who had different levels of exposure to risk and varying access to resilience-promoting resources (e.g., a supportive adult), about their experiences of service use. They found that adolescents at higher risk and with low resilience voiced a preference for professional support with more relaxed boundaries, such as contact outside of official therapy time, implying that this type of therapeutic relationship may be a protective factor for these adolescents (Ungar et al., 2018). On the other hand, adolescents with high resilience and at low risk described less need for professional support in general due to the social capital that they already had in their lives, implying that the social support networks that these adolescents already have access to may be protective enough without additional therapeutic support (Ungar et al., 2018).

Given the rising rates of mental health issues among adolescents in the UK (Vizard et al., 2020), recent UK government policy has moved towards schools being key sites from which to deliver interventions to promote wellbeing and prevent the onset of mental health issues (Department of Health and Social Care & Department for Education, 2018). The significant proportion of time that young people spend in school means that schools can reach a much wider range of young people than clinical services and can overcome barriers associated with attending clinical services, such as travel, timing, and cost issues (Masia-Warner, Nangle, & Hansen, 2006). As studies of resilience are inevitably contextually situated because what is experienced as protective in one context may not be available or seen as adaptive in another (Ungar, 2008), there is a need for qualitative research specifically in a UK context to explore the factors and processes that young people find to be protective in the face of difficulties in life, including how, why, and in what circumstances these may vary. Such findings can then be used to inform the development of effective school-based prevention and early intervention programmes to meet a range of needs.

Consequently, in the current study, we sought to build on existing understanding in this area by taking a qualitative approach to inquiry and exploring the factors, processes, and contexts (with a focus on the concepts of coping and social support) that are deemed protective from adolescents’ own perspectives and in their own words, within the setting of a school-based mental health prevention programme in the UK. Specifically, our study sought to address the following aims using qualitative methods: (1) To explore the coping strategies and sources of support that adolescents identify as protective (or not) in the face of difficult situations and feelings over a three-year period; (2) To examine how and why this may vary in line with the levels of adversity that they report experiencing in life.

## Method

### Research Design

We used an interpretive, qualitative research design to explore, through semi-structured interviews, young people’s lived experiences of and perspectives on problems and difficulties in daily life, coping strategies, and accessing or receiving support both from formal sources, including professionals, and informal sources, including family and friends. Our analysis primarily draws on Braun and Clarke’s (2006, 2021) guidance for conducting thematic analysis and is underpinned by a critical realist epistemological perspective. This takes the view that while there is a real world that exists independently of our perceptions and constructions of it, our understanding of it is a construction from our own point of view (Maxwell, 2010). This means that we see our analysis of the data as being an interpretation of participants’ reality, which we have constructed from our own perspectives, contexts, and views of the world. We are experienced researchers in the child and adolescent mental health research field, currently working in the context of evaluating interventions seeking to enhance young people’s resilience, mental health, and wellbeing, to learn about what helps to manage and prevent mental health difficulties.

### Setting for the Study

HeadStart is a six-year, school-based, mental health prevention programme, which launched in 2016 in six local authorities in England. The aim of HeadStart is to promote resilience, wellbeing, and positive mental health through the delivery of a range of preventive and early intervention approaches seeking to boost young people’s coping strategies and environmental resources (Evidence Based Practice Unit, 2018, 2019). A five-year qualitative longitudinal study is being conducted to explore young people’s experiences of HeadStart and, in doing so, examine the role and place of HeadStart more broadly within young people’s perspectives on coping and receiving support. Young people were invited to take part in the study by school staff or HeadStart staff if they had already received support from HeadStart by the first timepoint of the study or if they were identified as likely to receive it in future. To date, 82 interviews with the same cohort of young people have been conducted at Time 1 (2017 or 2018), 78 at Time 2 (2018 or 2019), and 55 at Time 3 (2019). Data collection in 2020 (Times 3 and 4) was paused due to Covid-19 restrictions.

### Ethical Considerations

Ethical approval for this study was granted by the University College London (UCL) Research Ethics Committee (ID number 7963/002). As all participants were under the age of 16, written informed consent was sought from the young people’s parents/carers and written assent to take part and for the publication of their anonymised data was sought from the young people at the outset of the study. It was made clear in study information sheets that participation was voluntary, and that participants could withdraw at any time without consequence. Participants received a £10 voucher after each interview as a thank you for taking part. To protect participant confidentiality, interview transcripts were anonymised (e.g., with names of people and places removed).

### Participants

A subsample of 31 participants from the wider qualitative longitudinal study sample was selected for inclusion in the present study. The subsample represented nine secondary schools across four of the HeadStart areas. Demographic information about the subsample can be seen in Table 1. All 31 participants had taken part in Time 1, 2 and 3 interviews, yielding a total subset of 93 interviews. 25 participants from the wider study sample were excluded from the subsample as they were missing interviews at Time 2 or 3. Given our study’s focus on adversity, 14 participants were excluded because they did not discuss coping strategies and support in the context of experiencing any mental health difficulties, family strain, or bullying, nor did they not report receiving any targeted support from HeadStart at Time 1. Targeted (indicated or selective) support is offered to select students, including those with mild or subclinical symptoms of a mental disorder or those with experience of particular risk factors, such as parental mental health issues (Campbell, 2004; Werner-Seidler, Perry, Calear, Newby, & Christensen, 2017). 12 participants from one HeadStart area were excluded because they were up to two years younger (age 9–10 years) than the majority of the young people (age 11–12 years) at Time 1, thus they did not align with our study’s focus on adolescence.

**Table 1:** Self-Reported Demographic Information about the Subsample (N = 31)

Demographic information	*N*
Sex	
Female	18 (58%)
Male	13 (42%)
Age^a^	
Time 1	11.08 to 12.09 years (*M* = 11.95, *SD* = 0.29)
Time 2	12.09 to 13.09 years (*M* = 12.85, *SD* = 0.39)
Time 3	13.05 to 14.11 years (*M* = 13.69, *SD* = 0.46)
Ethnicity	
White British	22 (71%)
Any other White background	4 (13%)
Mixed: White and Black Caribbean	2 (7%)
Mixed: White and Asian	1 (3%)
Black or Black British: African	1 (3%)
Any other Asian background	1 (3%)

^a^ Exact age data were missing for two participants at Time 2 and one participant at Time 3

### Data Collection

The interviews were conducted by four members of the research team (including the first and last authors). The interviews took place in a private room at participants’ schools. Where possible, the same researcher interviewed each participant at all three timepoints. All interviews were audio recorded and transcribed verbatim. The interviews in our subsample ranged in length from 20.47 to 60.05 min at Time 1 (*M* = 40.3, *SD* = 9.86), 21.39 to 68.43 min at Time 2 (*M* = 38.05, *SD* = 12.95), and 22.55 to 63.23 min at Time 3 (*M* = 41.83, *SD* = 11.16).

The interview schedule developed by the research team was semi-structured, which meant that while there were core questions asked by the researcher in each interview, the conversation around these key areas was led by participants’ responses. Core interview questions asked about participants’ experiences of and perspectives on coping with problems and difficult situations or feelings in life, including strategies that they drew on and social and professional support that they accessed (and their opinions on this). At Times 2 and 3, the interview schedule also asked about any changes over time in relation to topics raised previously. For example, ‘You mentioned when I met with you last year that you were having arguments with your friends, how are your friendships this year?’.

### Reflexivity

Reflexivity is a means for the researcher to critically engage with their role in the research process, including remaining self-aware and cognizant of their own influence on the research and in turn how the research may be affecting them (Probst, 2015). The research team designed an interview reflection tool to facilitate interviewers in debriefing following each interview. Reflections were audio-recorded and discussed further with the research team lead (the first author) when the interviewer deemed this to be helpful. The intention was to provide a space for interviewers to offload their immediate thoughts and feelings following each interview, and to encourage them to develop their interview skills through reflecting on their technique in each interview.

We reflect that our approach to data collection and analysis is inevitably influenced by our own understanding and experiences of the research area. For instance, our approach to asking young people about their experiences of coping and support was influenced by our theoretical grounding as researchers within systemic theories of resilience. Thus, in each interview, we specifically explored young people’s experiences within the context of key systems, including family, peers, and school. We also recognise that our approach to data collection and analysis is influenced and limited by our own understanding and experiences of the world, including sociodemographic differences between ourselves and the young people, such as in terms of age, ethnicity, and gender identity. For example, the age gap between ourselves and participants, in conjunction with the interviews taking place on school premises, could have reinforced hierarchical structures inherent in schools (Ozer, Newlan, Douglas, & Hubbard, 2013), and thus inhibited participants from speaking openly in their interviews about their experiences and opinions. Therefore, we endeavoured at each interview to establish a secure, non-hierarchical space for the young people to speak to us in, emphasising confidentiality (unless any safeguarding issues arose), young people’s right to withdraw at any time, and that there were no right or wrong answers. Our interview schedules were also developed in conjunction with young people to ensure that the questions were meaningful to and understood by our target audience.

### Data Analysis

To address our study aims, our analysis sought to answer two research questions sequentially: (1) What helps adolescents to manage difficult situations and feelings over a three-year period? (2) How does ‘what helps’ vary depending on the level of adversity that adolescents report experiencing in their lives over time?

To answer the first research question, a hybrid deductive/inductive thematic analysis was conducted by the first and second authors using NVivo (version 12) to identify the coping strategies and sources of support that participants reported drawing on at Times 1, 2, and 3. An existing thematic framework of young people’s coping behaviour was used to facilitate this, which was derived through an earlier inductive thematic analysis, guided by Braun and Clarke’s (2006) methodology, of all 82 interviews conducted at Time 1 with the young people taking part in the wider qualitative longitudinal study (see Stapley et al., 2020a). The framework consisted of the following main themes: Activities and strategies; Disengaging from problems; Standing up for yourself; Acceptance of problems; Social support; HeadStart support; Other professional support; Hiding feelings or problems (Stapley et al., 2020a).

We used this existing framework to guide our coding of the interviews in the present study, but also renamed and restructured themes, and created new themes, as necessary to best reflect the Time 1, 2, and 3 interview data. The coding process involved collating relevant transcript extracts under each theme. For instance, a new subtheme of ‘Support from boyfriends or girlfriends’ was developed from coding participants’ Time 2 and 3 interviews and included within a new main theme of ‘Support from close and trustworthy friends’. ‘Hiding feelings or problems’ ceased to be a main theme in the present study, as it became apparent when exploring the data across all three timepoints that this was typically spoken about in relation to particular groups of people, principally parents, friends, and school staff. Thus, in our study, participants’ references to finding it difficult to talk to or hiding problems or feelings from others have been captured as relevant when describing their experiences and perceptions of support from these groups.

To answer the second research question, an inductive thematic analysis was conducted, again by the first and second authors using NVivo (version 12), guided by the six steps outlined by Braun and Clarke (2006, 2021): becoming familiarised with the data; systematically coding the data or applying descriptive labels to transcript extracts; collating similar codes (labels) to generate initial themes; developing and reviewing themes; refining and giving names and definitions to themes; and the report. The interviews were re-coded in NVivo to develop new themes, which this time delineated the difficult situations and feelings that participants reported experiencing at Times 1, 2, and 3.

Braun and Clarke (2021) take a reflexive approach to thematic analysis, which can be distinguished from codebook or coding reliability approaches to thematic analysis. We view our analysis as primarily reflexive, but at times reflecting elements more akin to a codebook approach. Our use of an existing thematic framework, for example, when answering our first research question perhaps more closely reflects a codebook approach, whereby the themes were developed using the Time 1 dataset and then used to guide our analysis of the Time 2 and 3 datasets, with refinements made as necessary in light of new data. By contrast, the analysis process for our second research question took an entirely open and bottom-up approach to both coding and theme development, which aligns more closely with a reflexive approach.

The first and second authors worked together throughout the analysis for both research questions to code the data and develop themes, using a collaborative approach to facilitate rich, in-depth engagement with the data (Braun & Clarke, 2019), and to ensure that our interpretations remained grounded within the data. However, we did not seek to assess interrater reliability during our analysis, thus our analysis was not aligned with a coding reliability approach to thematic analysis. This is because, in line with Braun and Clarke’s (2021) reflexive approach, we view researcher subjectivity as a *“resource for knowledge production which inevitably sculpts the knowledge produced, rather than a must-be-contained threat to credibility”* (p. 334–335), thus interrater reliability is not seen as a marker for quality of analysis.

Braun and Clarke (2021) also distinguish between themes defined as patterns of shared meaning organised by a central concept, which is a core part of their reflexive approach, and themes defined as summaries of participant responses in relation to particular topics within the data, which is more aligned with a codebook approach. Researchers taking a reflexive approach to thematic analysis need to justify their use of the latter (Braun & Clarke, 2021). Due to the large volume of data that we were working with and our aim of drawing relatively broad, concrete comparisons between groups of participants, we reflect that some of our themes align more closely with what Braun and Clarke (2021) describe as ‘shared-topic’ themes (e.g., ‘Varying trajectories of HeadStart and other professional support’), rather than ‘shared-meaning’ themes (e.g., ‘Disengaging from difficulties’).

As the final step in our analysis, by examining the transcript content coded to each theme delineating the difficult situations and feelings that each participant reported experiencing at each timepoint, participants were then divided into three groups by the first and second authors, each representing a different level of adversity. The three groups were: Group A - participants who reported that their levels of difficulty in life had improved or were manageable by Time 3; Group B - participants who reported experiencing some ongoing difficulties and some areas of improvement by Time 3; Group C – participants who reported that their levels of difficulty had deteriorated or were hard to manage by Time 3. The authors initially separately allocated each participant to one of the three groups and then checked each other’s allocations, with discussion of any instances of disagreement until agreement was reached.

## Findings

**Fig. 1:**
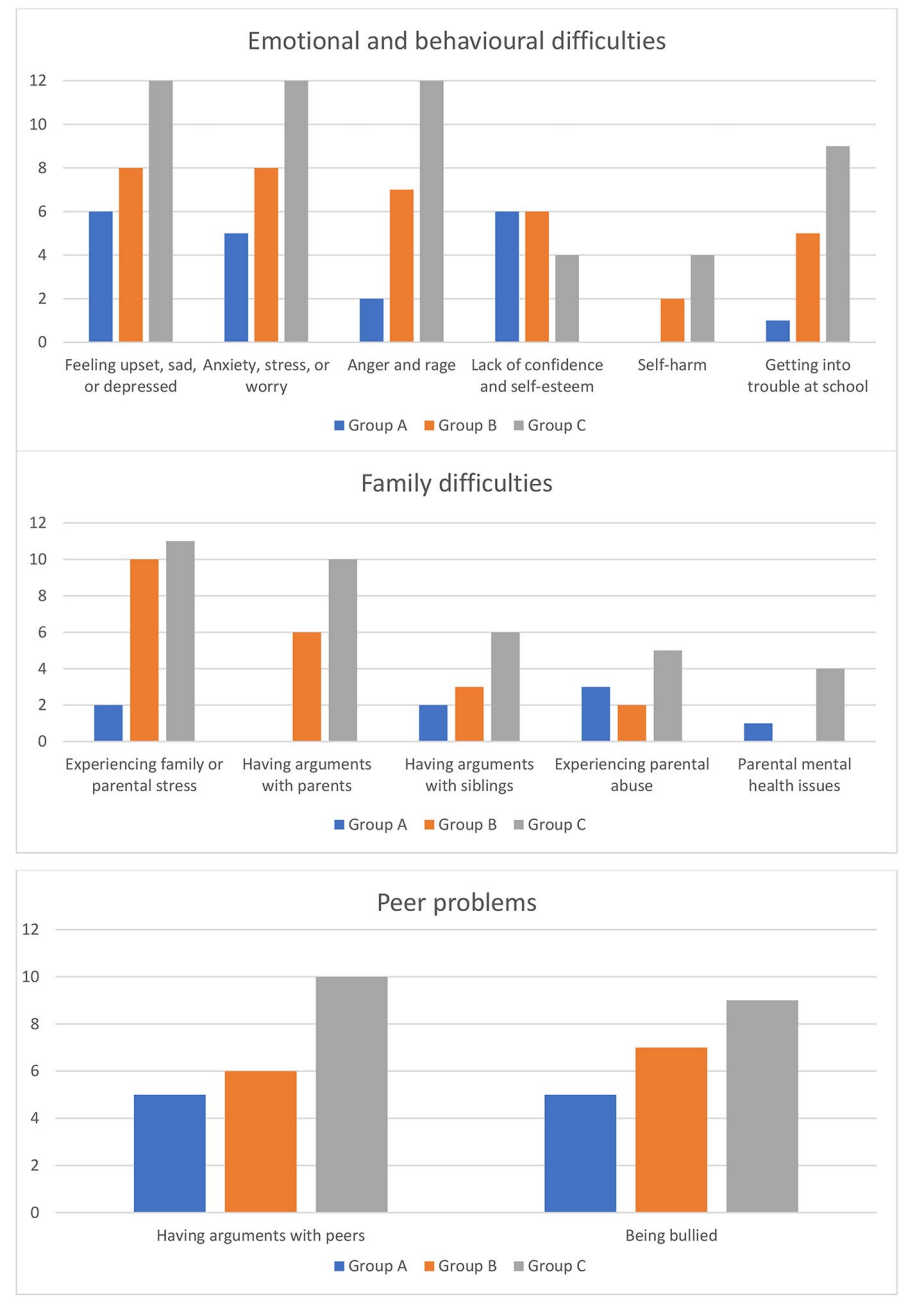
Frequencies (*N*) of participants in each group who reported experiencing particular difficult situations and feelings at any timepoint

Table 2; Fig. 1 show the difficult situations and feelings reported by participants in each of the three groups at any timepoint.

**Table 2:** Frequencies (N) and Proportions (%) of Participants in each Group who Reported Experiencing Particular Difficult Situations and Feelings at any Timepoint

	Group		
	Group A (*N* = 8)	Group B (*N* = 11)	Group C (*N* = 12)
Difficult situations and feelings	*N* (%) participants	*N* (%) participants	*N* (%) participants
Emotional and behavioural difficulties			
Feeling upset, sad, or depressed	6 (75%)	8 (73%)	12 (100%)
Feelings of anxiety, stress, or worry	5 (63%)	8 (73%)	12 (100%)
Anger and rage	2 (25%)	7 (64%)	12 (100%)
Lack of confidence and self-esteem	6 (75%)	6 (55%)	4 (33%)
Self-harm	0	2 (18%)	4 (33%)
Getting into trouble at school	1 (13%)	5 (45%)	9 (75%)
Family difficulties			
Experiencing family or parental stress	2 (25%)	10 (91%)	11 (92%)
Having arguments with parents	0	6 (55%)	10 (83%)
Having arguments with siblings	2 (25%)	3 (27%)	6 (50%)
Experiencing parental abuse	3 (38%)	2 (18%)	5 (42%)
Parental mental health issues	1 (13%)	0	4 (33%)
Difficulties with peers			
Having arguments with peers	5 (63%)	6 (55%)	10 (83%)
Being bullied	5 (63%)	7 (64%)	9 (75%)

As can be seen in Table 2; Fig. 1, comparatively high proportions of participants across the three groups reported experiencing feelings of sadness and anxiety, as well as experiences of being bullied at any timepoint. However, Group C contained the highest proportions of participants who reported experiencing difficulties with anger, self-harm, arguments with parents and/or siblings, parental abuse, parental mental health issues (such as depression), getting into trouble at school, and arguments with peers. By contrast, the highest proportion of participants who reported lacking in confidence and self-esteem could be seen in Group A. Groups B and C contained the highest proportions of participants who reported experiencing some form of family or parental stress (such as animosity between parents or family financial difficulties) at any timepoint.

**Fig. 2:**
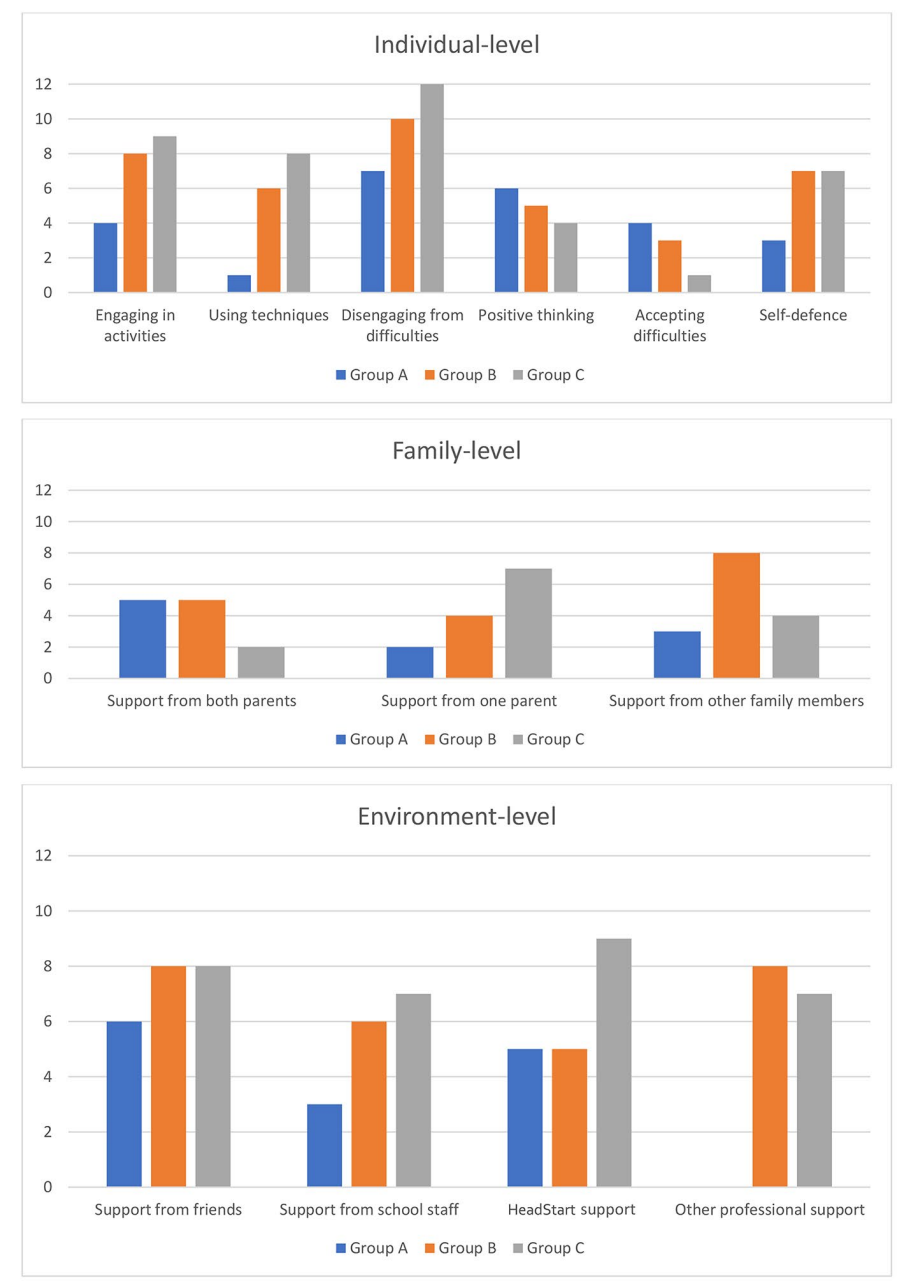
Frequencies (*N*) of participants in each group who reported drawing on particular coping strategies and sources of support at two or more timepoints. (Note. Current or historic targeted support received from HeadStart or other professionals (e.g., CAMHS is shown as reported at any timepoint by participants)

Table 3; Fig. 2 present the coping strategies and sources of support (organised in terms of individual-, family-, and environment-level protective factors and processes) that participants across the three groups reported drawing on at two or more timepoints to manage difficulties in life. Reports at two or more timepoints was considered a proxy for participants’ consistency in usage of specific coping strategies and sources of support over time. Previous quantitative longitudinal research has identified stability in adolescents’ reports of using particular coping strategies over at least a two-year period (Valiente, Eisenberg, Fabes, Spinrad, & Sulik, 2015).

**Table 3:** Frequencies (N) and Proportions (%) of Participants in each Group who Reported Drawing on Particular Coping Strategies and Sources of Support at Two or More Timepoints

	Group		
	Group A (*N* = 8)	Group B (*N* = 11)	Group C (*N* = 12)
Coping strategies and sources of support	*N* (%) participants	*N* (%) participants	*N* (%) participants
Individual-level factors and processes			
Engaging in activities	4 (50%)	8 (73%)	9 (75%)
Using techniques	1 (13%)	6 (55%)	8 (67%)
Disengaging from difficulties	7 (88%)	10 (91%)	12 (100%)
Positive thinking	6 (75%)	5 (45%)	4 (33%)
Accepting difficulties	4 (50%)	3 (27%)	1 (8%)
Self-defence	3 (38%)	7 (64%)	7 (58%)
Family-level factors and processes			
Support from both parents	5 (63%)	5 (45%)	2 (17%)
Support from one parent	2 (25%)	4 (36%)	7 (58%)
Support from other family members	3 (38%)	8 (73%)	4 (33%)
Environment-level factors and processes			
Support from friends	6 (75%)	8 (73%)	8 (67%)
Support from school staff	3 (38%)	6 (55%)	7 (58%)
HeadStart support^a^	5 (63%)	5 (45%)	9 (75%)
Other professional support^a^	0	8 (73%)	7 (58%)

^a^ Current or historic targeted support received from HeadStart or other professionals (e.g., child and adolescent mental health services; CAMHS) is shown as reported at *any* timepoint by participants

### Individual-level Factors and Processes

#### Engaging in Activities

Participants described engaging in different activities (e.g., playing video games, drawing, and playing football) to take their mind off their problems, have fun, or relax: *“When I’m thinking about the worries and when I’m, like, drawing, it’s, like, makes me a lot, like, do you know, thinking about the worries, it makes them go somewhere else”* (Group A, Time 2). The prevalence of this theme, in terms of references at two or more timepoints, was higher in Groups B (73%) and C (75%) than Group A (50%).

However, participants in Groups B and C also reported that engaging in activities did not always help. Reasons for this included that some problems (such as a grandparent dying) can make you feel so sad that engaging in an activity does not help, some activities (e.g., boxing) can make you feel angrier instead of calmer, and some activities (e.g., eating comfort food) are not necessarily good for you: *“I realised me doing boxing has made me more angry and then, then when people are annoying me, then I know that I have the power to do something”* (Group C, Time 3).

#### Using Techniques

Participants described using different techniques or specific strategies (e.g., deep breathing techniques, stress balls, and counting to 10), sometimes suggested by a professional, to try to regulate their emotions: “*When I was clicking my fingers I always… I just, when I got nervous or I got angry or something like that, I feel like that calmed me down”* (Group C, Time 3). The prevalence of this theme, in terms of references at two or more timepoints, was higher in Groups B (55%) and C (67%) than Group A (*N* = 13%).

Yet, participants in Groups B and C also reported limitations in the efficacy of strategies, such as forgetting to take deep breaths to manage their anger in the heat of the moment. Participants in Groups B (18%) and C (33%) also mentioned engaging in self-harm as a coping strategy at various points in their lives. However, self-harm was only identified as a current coping strategy by the third timepoint by participants in Group C: *“[My sister] just tells me I’m an idiot, (chuckles) and I need to stop doing it”* (Group C, Time 3).

#### Disengaging from Difficulties

Almost all participants across the three groups described instances at two or more timepoints when they had dealt with problems by deliberately disengaging from them, such as through distracting themselves, forgetting problems, choosing to put problems out of their mind, or ignoring the existence of problems and individuals who were upsetting them (e.g., bullies): *“I just try my best to not listen to them and just ignore them”* (Group A, Time 2).

#### Positive Thinking

Participants described engaging in positive thinking in the face of difficulty, including trying to see the positive side of difficult situations, thinking positive thoughts to cheer themselves up, and persevering and not giving up: *“Make something happy out of it or just think about generally something that makes you happy and then like… sort of like post the angry feelings out with the happy feelings”* (Group B, Time 1). The prevalence of this theme, in terms of references at two or more timepoints, was highest in Group A (75%), as compared to Groups B (45%) and C (33%).

#### Accepting Difficulties

Participants described how over time they had become used to difficult situations or had simply accepted the existence of particular aspects of life that they found hard, which could eventually make such situations less stressful and easier to handle: *“I was really shy, and like, I was scared to talk to other people, I kind of got used to it and, like, I’m not as shy anymore”* (Group A, Time 3). This theme also included participants’ references to waiting for problems or difficult feelings to pass or ‘blow over’. The prevalence of this theme, in terms of references at two or more timepoints, was higher in Groups A (50%) and B (27%) than Group C (*N* = 8%).

#### Self-defence

Participants described situations (principally arguments with friends, family members, or teachers) at two or more timepoints that in their view required them to challenge unwanted behaviour from others or defend themselves (verbally or physically): *“I ain’t just going to stand there and have everyone call me a wimp when they hit me, and I don’t hit them back. I’m just going to stand there and hit them back”* (Group B, Time 1). The prevalence of this theme was higher in Groups B (64%) and C (58%) than Group A (38%).

### Family-level Factors and Processes

#### Parents as a Source of Comfort and Advice

Participants in Group A often referred to both of their parents (63%) as being a supportive presence in their lives: *“The first people I would go to are my parents if there was a problem. Which is really good, and they would give me their honest opinion”* (Group A, Time 2). This included feeling able to and wanting to talk to their parents about their problems, with reference to their parents making them feel better, giving them advice, or helping them to see another perspective or reach a solution. Similarly, 45% of participants in Group B described both of their parents, at two or more timepoints, as being a source of support, comfort, and advice in difficult situations. A higher proportion of participants in Group C identified one of their parents (58%), usually their mother, as being a supportive presence in their lives, as opposed to both parents (17%). This parent was described as being a source of advice and comfort.

#### Parents at Arms-length

Only a minority (25%) of Group A participants perceived one parent as being a more prominent source of support than the other at two or more timepoints. Both of these participants self-identified as female and described feeling more able to talk to their mothers about problems than their fathers, who they felt may not understand their problems to the same degree that their mothers would: *“If it’s to do with girls or problems at school, I probably wouldn’t necessarily speak to [my dad] about it but sometimes, I do”* (Group A, Time 2). Similarly, participants in Group B (36%) who described one parent as being a more prominent source of support than the other indicated that they had a closer relationship with one parent (usually their mother). By contrast, the other parent for participants in Group C was often seen as being a source of difficulty in their lives or as less available to talk to (such as because they were busy or they did not live with them), and so was considered to be a less suitable source of support for these reasons.

Participants in Group B also described instances of not always feeling able to, not always wanting to, or hesitating to talk to their parents about their problems. For instance, if they thought that they might worry or upset their parents, if their parents were not available to talk to, if they thought that a problem was not major enough to warrant talking to their parents about, or if, in general, they preferred trying to resolve problems on their own first. Similarly, participants in Group C described finding it hard to speak to their parents about some issues, such as feeling sad or having low self-esteem, because, for example, they felt that their parents did not understand what they were going through.

*My mum is always like, ‘Toughen up’. I literally can’t and like I don’t know what to say to my mum when she says to me, ‘Toughen up’, when she’s like, ‘You need to stop crying, you need to grow up’, and I don’t know if I can.* (Group C, Time 3)

#### Other Family Members as a Supportive Presence

Participants also described drawing on support from other members of their families. The prevalence of this theme, in terms of references at two or more timepoints, was higher in Group B (73%) than Groups A (38%) and C (33%). There were participants in all three groups who saw their siblings (and also, in a small number of cases, their cousins) as ‘having their back’ and as being someone to talk to about problems and seek advice from because, for example, they had had similar experiences to each other: *“If there’s any problems with me, like, s- I, I could talk to [my sister]. And like, she’ll listen. Like, I’ll, I can trust her […] she won’t, like, tell my mum if I don’t want her to”* (Group B, Time 2). In terms of support from extended family, participants across the three groups most often referred to their grandmother as a source of support, describing them as another person to talk to about problems and seek advice from, in the absence of or in addition to parental support. Participants in Groups B and C also described their pets as being a source of comfort and as cheering them up when they were feeling sad, worried, or angry.

### Environment-level Factors and Processes

#### Support from Close and Trustworthy Friends

Similar proportions of participants across Groups A (75%), B (73%), and C (67%) described at two or more timepoints how their friends (including, for a minority, boyfriends or girlfriends) were a source of support in times of difficulty. Friends were referred to as cheering you up, standing up for you in arguments or against bullies, and being someone to talk to and receive relatable advice from, for example for problems that your parents would not understand. However, while trust in family members was more implicit, there were participants across all three groups who mentioned having specific or close friends whom they trusted more than others to keep their problems confidential: *“I have one friend […] she’s like really… we talk about everything. When I told, when I say something to her, it then doesn’t come out anyone’s mouth”* (Group B, Time 3).

#### School Staff as a Double-edged Sword

Higher proportions of participants in Groups B (55%) and C (58%), as compared to Group A (38%), reported drawing on or being given support, when needed, from school staff (teachers and/or pastoral care staff) at two or more timepoints. Participants in Group A primarily described school staff as mediating in situations of bullying or arguments with peers, and felt that particular school staff members were supportive or were there for them to talk to if they needed to. However, Group A participants also reported that generally they felt more comfortable seeking support from family and friends, although they would consider talking to a school staff member if a problem was really serious: *“If we’re talking about like school, no, not really, because um I just feel like that’s, that’s not what I do, that’s not how I deal with things. Like, I, I, I’d rather go to my friends or my mum”* (Group A, Time 3).

Participants in Group B similarly described school staff as intervening in difficult situations with peers, and also described seeking support from specific school staff members if they were upset or if they wanted someone to talk to. However, Group B participants also mentioned times when school staff had not always been able to provide effective support. For instance, school staff were not always available to talk to about problems, they did not always listen or take action, or they could not always be trusted to keep problems confidential. Talking to a teacher about issues with peers could also result in you being labelled as a ‘snitch’, which was not helpful: *“If I do tell on the people who do it, they w- they will A, start calling me a snitch, and B, start making fun of [me] even more”* (Group B, Time 1).

Participants in Group C described having arguments with and feeling blamed by teachers, but also described instances when they had been given support by particular members of school staff, including seeing them as someone to speak to about difficult family situations, bullying, or managing anger. However, Group C participants also described times when they had struggled to trust school staff, including having an awareness that there may be consequences of speaking to school staff (such as an investigation happening), worries about teachers forming an opinion of you, and experiences of or anticipation of not feeling understood by school staff: *“I find it a bit difficult to tell teachers because I know that their policy is obviously they can’t tell pupils, but they can tell like people if it’s a major problem like anyone [is] in danger”* (Group C, Time 3).

#### Varying Trajectories of HeadStart and Other Professional Support

Group C contained the highest proportion of participants who reported receiving targeted support from HeadStart at any timepoint (75%), followed by Group A (63%) and Group B (45%). On the other hand, Group B contained the highest proportion of participants who reported receiving current or historic support from other professionals (outside of HeadStart) at any timepoint (73%), followed by Group C (58%) and Group A (*N* = 0).

At Time 1, four participants in Group A reported meeting with a peer mentor (an older student at school). They described the positive impact of this type of HeadStart support, including learning coping strategies, having someone relatable to talk to, and boosting their confidence. At Time 2, none of these participants reported still being in receipt of peer mentoring. Three had been offered additional HeadStart support (such as involvement in co-producing their area’s programme). However, one had decided not to take part as none of her friends had signed up this year, another’s support had stopped because of school staff strikes, and the other participant’s support had never begun. One participant in Group A mentioned receiving HeadStart support for the first time at Time 2 (counselling). At Time 3, no participants in Group A reported receiving any HeadStart support: *“I just stopped it because I didn’t think I’d need it anymore”* (Group A, Time 3).

At Time 1, four participants in Group B reported receiving HeadStart support, including one-to-one (peer mentoring or counselling) and small group-based support (psychoeducational sessions or co-production meetings). They described receiving useful advice about coping with being bullied and handling difficult feelings (such as anger and anxiety), enjoying being involved in HeadStart, and finding it helpful to have someone to speak to about their worries.

*They give some really good ad- advice, like when we was learning about worrying and stress, there was like some stuff that we can do to like help deal with that, and then things that we do, like, that are maybe bad and like how we can stop that like happening, and like a better way to cope with it.* (Group B, Time 1)

At Times 2 and 3, only one participant in Group B was still receiving HeadStart support. This participant reported feeling more confident and less anxious as a result, but also felt that some of their group sessions had been disrupted by other students misbehaving. Two participants in Group B did not feel at Times 2 and 3 that they needed support from HeadStart anymore, as they were feeling better. However, two other participants (one of whom also described receiving ongoing support from a professional at CAMHS to manage her anxiety across Times 1, 2, and 3, and the other of whom mentioned seeing a school counsellor at Time 2) stated that they would like to receive support from HeadStart again at Time 2. One of these participants still felt the same at Time 3, whereas the other felt that they did not need any support from HeadStart by Time 3.

Two participants did not report receiving support from HeadStart at any timepoint, but did mention taking medication to manage attention deficit hyperactivity disorder (ADHD) across Times 1, 2, and 3. Five participants also described historic contact with social services, counselling, and/or therapy to manage such issues as school-related stress or difficult family situations. Four of these participants identified aspects of this support that had been unhelpful, such as finding it boring, finding it hard to talk about difficult feelings or situations, or having their trust betrayed. Only one of these participants stated that his therapy had had a positive impact on his levels of worry and stress at the time. However, he also said that he would not necessarily want to receive therapy again.

*Nobody wants to be the person who’s, like, gone to therapy three years in a row. And um ‘cause I don’t want to miss school as well because last time I had to go to therapy I, I, I missed a lot of school.* (Group B, Time 2)

At Time 1, five participants in Group C reported receiving one-to-one (peer mentoring) or small group-based HeadStart support (psychoeducational sessions or co-production meetings). Participants described getting things off their chests through talking to others about their problems, and learning how to manage their worries and anger. One of these participants also reported receiving ongoing small group and one-to-one support from HeadStart support workers across Times 2 and 3. However, the other four participants reported no longer receiving HeadStart support at Time 2 because it had ended or because they had not found it helpful.

Of the latter four participants, one participant did not report receiving any HeadStart support at Time 3 either. Another reported receiving HeadStart support again at Time 3 in the form of co-production meetings, as well as having contact with social care and a school counsellor, which he described as limited in its utility. The remaining two participants described receiving support from statutory CAMHS, social care, and/or a counsellor instead of HeadStart at Time 2. Both felt that this support was more helpful. By Time 3, one of these participants was still receiving ongoing counselling, and the other had stopped receiving support from statutory CAMHS, but had been referred to another form of small group-based HeadStart support at school.

***Why do you think the CAMHS course has been more helpful than [HeadStart]?*** *They explained it more in detail and like, I don’t know. Talking to like other people with ADHD and stuff and I found than better than. ‘Cause like not really much people has ADHD in this school.* (Group C, Time 2)

Two participants in Group C reported receiving HeadStart support for the first time at Time 2 (counselling). For one of these participants, this support had continued at Time 3, although with a new counsellor, as her previous counsellor at Time 2 had not managed to help her. For the other participant, this support (which had also included therapeutic work with her parents) had ended by Time 3. However, both of these participants also mentioned receiving support from statutory CAMHS in relation to feelings of anxiety, depression, and self-harm at Time 3.

*[My previous counsellor] couldn’t cope with the situation. It was too hard for her to deal with because, she, she, she was too young […] like, she couldn’t help, she didn’t know what to do with it. Um, and that’s why we had to go with a different person.* (Group C, Time 2)

Two participants in Group C reported receiving HeadStart support (e.g., online counselling) for the first time at Time 3. One of these participants also mentioned receiving professional support at Time 1 for ADHD. The other participant mentioned historic contact with social care at Time 2 and current support from social care at Time 3. This participant described having recently been referred to a youth worker by her social worker for additional emotional support, which she felt had been helpful.

## Discussion

Our sample consisted of adolescents who were attending schools in England implementing a mental health prevention programme, HeadStart. Within our sample, we identified three groups of participants: those who reported that their levels of difficulty in life had improved or were manageable by the third year of the study (Group A); those who reported experiencing some ongoing difficulties and some areas of improvement (Group B); and those who reported that their levels of difficulty had deteriorated or were hard to manage (Group C). Young people who reported experiencing higher and/or persistent levels of difficulty in life over time, as compared to their counterparts, more often described using such coping strategies as self-defence and self-harm, referred to limitations in the efficacy of particular activities and strategies, voiced reasons why they were reluctant or unable to seek support from their parents, perceived limitations in support from school staff, and reported more mixed experiences of support from professionals, in terms of the timing of support and their perceptions of its efficacy. This aligns with findings from a previous qualitative study conducted to examine change over the first two years of HeadStart in young people’s experiences of difficulties and support, drawing on the wider qualitative longitudinal study sample of 78 participants (Stapley, Eisenstadt, Demkowicz, Stock, & Deighton, 2020b). This study found that young people who described having more difficult experiences in general over the two-year period were more likely to report having sources of support characterised by uncertainty or ambiguity (Stapley et al., 2020b).

The findings of the current study also reflect previous quantitative findings, which have similarly identified variation in the incidence and impact of protective factors according to the level of adversity that young people are experiencing (e.g., Fergusson, Lynskey, & Horwood, 1996; Kassis et al., 2013). However, our qualitative findings also add to this previous quantitative research by showing when, how, and why particular factors and processes may be more or less protective from the perspective of young people who are experiencing varying levels of adversity. For instance, in previous research, friendships have been found to mitigate against the negative effects of bullying (Kendrick, Jutengren, & Stattin, 2012), and family adversity (Criss, Pettit, Bates, Dodge, & Lapp, 2002). Yet, while comparatively high proportions of participants across all three groups in our study referred to their friends as a source of support in times of difficulty, the proportion of participants who also described having arguments with their friends was highest in Group C. This could suggest that the quality of support may influence the level of protection that it can offer. Indeed, high quality friendships, defined in terms of perceptions of supportiveness, have been found to predict lower levels of future victimisation by bullies (Kendrick et al., 2012).

Quality may also be relevant when considering the limitations in the efficacy of particular coping strategies that participants in Groups B and C reported, as well as the use of self-harm as a coping strategy in a minority of cases. The coping strategy of positive thinking, on the other hand, employed by a majority of participants in Group A, has been identified in previous research as being an individual-level protective factor implicated in promoting young people’s resilience (Masten & Barnes, 2018), and as an adaptive coping strategy (Losoya, Eisenberg, & Fabes, 1998; Zimmer-Gembeck & Skinner, 2011). Yet, disengagement or withdrawal from problems has been found in previous studies to be associated with poorer mental health outcomes (e.g., (Seiffge-Krenke, 2004; Seiffge-Krenke & Klessinger, 2000). By contrast, our findings indicate that this is a strategy that the majority of young people engage in, regardless of their levels of difficulty in life (see also Stapley et al., 2020a). Perhaps this alternatively reflects previous findings from the emotion regulation literature that the use of distraction can enhance adolescents’ levels of positive affect and reduce their levels of negative affect, which may be a solution in the short-term (Wante, Van Beveren, Theuwis, & Braet, 2018).

While parental support was drawn on by young people in all three groups in our study, the majority of participants in Group C cited one parent, rather than both, as a source of support, with the non-supportive parent described as less available to talk to because for example, they were busy, they did not live together, or they were a source of difficulty in their lives. By contrast, the majority of participants in Group A referred to both of their parents as being a supportive presence in their lives. Previous qualitative studies have similarly highlighted the importance, from young people’s perspectives, of familial support in protecting against adversity or promoting recovery from mental health issues (e.g., Las Hayas et al., 2016; Smokowski & Reynolds, 1999). Indeed, close caregiver-child relationships have frequently been identified as a key family-level protective factor for young people in the face of adversity (Masten, 2021). The higher levels of familial stress reported by young people in Groups B and C, as compared to Group A, may explain the differences in the levels of familial support that they reported. For example, previous research has identified a negative association between interparental conflict and parental emotional support provision for young people (Riggio, 2004).

In terms of support from HeadStart, 61% of participants reported receiving some form of targeted HeadStart support by the end of the three-year period of our study: three-quarters of participants in Group C, just under half of Group B, and just under two-thirds of Group A. In Groups A and B, the majority of participants reported receiving support from HeadStart at Time 1 only. By contrast, in Group C, participants described a range of interactions with HeadStart, with some participants only reporting receiving support at one timepoint and others reporting receiving multiple forms of support across or at different timepoints. Our findings suggest that more long-term, regular, or sustained preventive intervention may be needed for young people who are experiencing higher levels of difficulty in life (see also Stapley et al., 2020b), such as those within Groups B and C, with perhaps more ‘light touch’ engagement for those experiencing less difficulty over time, such as those within Group A. The latter reflects Ungar et al.’s (2018) finding that adolescents with high resilience and low risk describe less need for professional support in general, potentially due to the social support that they already have.

School staff nominations are often a starting point for the identification of students for targeted interventions (Campbell, 2004). However, research has shown that teachers have less accuracy in identifying young people with emotional problems, compared to behavioural problems (e.g., Cunningham & Suldo, 2014; Splett et al., 2020), and with moderate or subclinical levels of symptoms, compared to severe (Splett et al., 2019). This could offer a potential explanation for why just under 50% of participants in Group B, for example, reported ever receiving HeadStart support, and why, for participants in Group C, the timing of their interactions with HeadStart varied. Thus, instating a regular wellbeing and mental health symptom check-in (such as using standardised self-report outcome measures) with young people each school year, and at the end of support interventions, could help to ensure that young people are offered additional support as and when it is needed (Humphrey & Wigelsworth, 2016; Stapley et al., 2020b).

On the other hand, it is possible that some participants were offered support and chose not to engage with it. Indeed, participants in Groups B and C identified both positive elements and limitations of the HeadStart and professional support that they had received, and described ways in which school staff could be supportive, but also voiced concerns about trusting school staff, or instances of not feeling listened to or understood by school staff. Previous qualitative studies of young people’s help-seeking behaviour have similarly identified young people’s perceptions of issues around school staff trustworthiness and availability as barriers to help-seeking (Helms, 2003; Lindsey & Kalafat, 1998). Such concerns could thus present a barrier to young people’s engagement with preventive interventions led by trained school staff or implemented within a school setting. Therefore, reviews of evaluations of existing programmes have highlighted the important role that a programme component focusing on promoting a supportive school environment or ethos can have in maximising engagement with and the effectiveness of school-based prevention and early intervention programmes (Weare & Nind, 2011).

Training in coping and problem-solving skills is often a key component in psychological interventions (Horwitz, Opperman, Burnside, Ghaziuddin, & King, 2016). Some of the coping strategies that participants described appear to align with treatment components across a range of evidence-based prevention and treatment approaches; for instance, positive thinking echoes aspects of cognitive restructuring activities within cognitive behavioural therapy (CBT) approaches (Clark, 2013). Yet, interventions that primarily aim to effect change at the level of the individual may have more limited efficacy for those who are experiencing high levels of contextual stress. Indeed, higher levels of family dysfunction have been found to predict poorer mental health treatment outcomes for adolescents (Phillips et al., 2000). This could explain why participants in Group C, approximately 90% of whom reported experiencing various sources of familial stress, were experiencing difficulties with their mental health and relationships by Time 3, despite 75% of them reporting receipt of HeadStart support by that point. Thus, following a review of resilience research, Luthar (2015) concluded that to maximise the potential for success, resilience-enhancing interventions should focus on invoking change in both the child and in their wider environment. For instance, the UK-based Thrive Framework is a needs-based approach to mental health and wellbeing support, which *“provides a set of principles for creating coherent and resource-efficient communities of mental health and wellbeing support for children, young people and families”* (Wolpert et al., 2019, p.2).

In a review of school-based mental health services, Rones and Hoagwood (2000) found that effectiveness was associated with multi-component programmes that targeted the ecology of the child, such as through involving parents (e.g., in parenting skill development sessions) and teachers (e.g., in classroom management techniques training). Similarly, in a systematic review, Weare and Nind (2011) found that the involvement of parents was cited in multiple reviews as a key ingredient in school-based preventive interventions. However, only one participant in our study mentioned receiving a HeadStart intervention that involved therapeutic work with their parents. Thus, particularly for young people who are experiencing higher levels of adversity in life (e.g., familial strain), our findings suggest that mental health prevention programmes like HeadStart could benefit from placing emphasis on implementing interventions that seek to effect change and boost the resources available within young people’s wider contexts, as well as within young people themselves. This reflects theories of resilience that emphasise the role of the individual’s connections and relationships with external systems in promoting resilience, as well as their own capacity to cope (e.g., Masten & Barnes, 2018; Ungar et al., 2008).

### Strengths and Limitations

Our study illuminates the different coping strategies and sources of support that adolescents experiencing varying levels of adversity in life view as protective (or less so) in relation to handling difficult situations and feelings over a three-year period – and why. A limitation of our study relates to the transferability of our findings. Most notably, our sample consisted of adolescents who were identified and invited to take part by school staff or HeadStart staff, based on current or potential future engagement in some aspect of HeadStart. Thus, our findings may overlook wider experiences, including those experiencing adversity without the school’s awareness, whose experiences of coping and social support may well be different. Similarly, there may be individuals who declined to take part, and we do not know how their experiences relate to those reported here.

Furthermore, our sample includes only those who chose to take part in all three interviews over the three-year period of the study. We do not know whether additional themes would be identified from interviews with adolescents who were unable to take part in all three interviews, such as if they had moved to a different school and were uncontactable by the research team. It is possible that the latter may be those who are experiencing particularly high levels of adversity. In terms of demographic information, we note that the majority of our sample identified themselves as being from a White ethnic background. Future research would benefit from an emphasis on sociodemographic representativeness in sampling, including direct exploration of how ethnicity may play a role in the protective factors and processes identified by adolescents in the UK. We note too that our findings are by nature specific to England, but may nevertheless offer value to researchers in other countries when considered in conjunction with research specific to their locality.

The findings solely reflect participants’ reports of experiences of difficulties in life, coping, and engagement with support that they remembered to or chose to share in their interviews. While every effort was made to help participants to feel comfortable and secure in the interview situation, including building rapport during each interview and ensuring where possible that the same researcher interviewed the same participant across all three timepoints, some participants may have felt less comfortable about sharing their experiences with a stranger, or sharing experiences that might have led to them feeling upset or embarrassed in their interviews (Docherty & Sandelowski, 1999). Lack of reference in an interview is not an objective indication that a participant definitely did not draw on a particular coping strategy or support source. For this reason, we did not seek to explore change over time in the minutiae of young people’s usage of particular coping strategies and support. Participants were also not explicitly asked about change over time in relation to each individual coping strategy and source of support mentioned in each interview.

The interview questions focused on participants’ experiences of coping and seeking or receiving support over each year of the study. Thus, it is important to note that while a broad range of protective factors have been identified in resilience research, including for example ‘skilled parenting’ and ‘connections with well-functioning communities’ (Masten & Barnes, 2018), our study focused specifically on the types of coping strategies and sources of support that young people report as being protective in the face of difficulty, as this was the focus of the interviews. In addition, there were a minority of problems (e.g., physical health issues) and sources of support (e.g., support from adults outside of the family and school) that were referenced so infrequently and by such a small number of participants that they were not included in our final list of themes.

Participants were grouped in our analysis based on their subjective experiences of the levels of difficulty in their lives that they were experiencing by the third timepoint of the study. We are unable to report objectively on the levels of mental health difficulties that would meet clinical thresholds within our sample. However, we reflect on the possible circularity of grouping participants in this way, in that individuals with higher levels of mental health concerns may be more likely to perceive situations as stressful or notice stressful aspects of their environment, or individuals experiencing more stressful situations or situated within a more stressful environment may be more likely to experience higher levels of mental health concerns.

## Conclusions

Our findings add to previous research by showing that the types, quality, and consistency of reported coping strategies and support, as described by adolescents in a UK context, varies in line with whether adolescents report experiencing higher or lower levels of adversity in life over time, and according to the resources that they have available within their physical and social environments. Future research in this area could qualitatively explore the additional factors and processes, both internal and external to the individual, beyond coping strategies and sources of support, that adolescents in this context describe as protective, and examine how these may also vary in line with the level of adversity experienced. Future research could also seek to further disentangle the differences between the presence and quality of different support sources and coping strategies as protective factors. Understanding the specific support and coping processes that are perceived to be most helpful by adolescents could indicate important areas for intervention.

Our findings suggest that more long-term, regular, or sustained early intervention may be needed for young people experiencing higher levels of difficulty in life. School staff and practitioners implementing regular reviews with young people regarding their support needs and preferences could help to ensure that young people receive timely support that is best suited to their needs. This aligns with a needs-based approach to providing support for young people’s mental health and wellbeing. Finally, for maximum effectiveness with young people who are experiencing high levels of contextual adversity, preventive interventions could benefit from being multi-component, such as incorporating family, school, and individual elements to boost the resources available within young people’s wider contexts, as well as within young people themselves.

## Data Availability

Access to data is restricted to the HeadStart Learning Team to comply with the study’s ethical approval. Materials (e.g., interview schedules) are available upon request to the corresponding author.
